# Relative growth and morphological sexual maturity size of the freshwater crab *Trichodactylus borellianus* (Crustacea, Decapoda, Trichodactylidae) in the Middle Paraná River, Argentina

**DOI:** 10.3897/zookeys.457.6821

**Published:** 2014-11-25

**Authors:** Verónica Williner, María Victoria Torres, Débora Azevedo Carvalho, Natalia König

**Affiliations:** 1 Instituto Nacional de Limnología (CONICET-UNL). Ciudad Universitaria Paraje El pozo s/n. Santa Fe. Argentina; 2 Facultad de Humanidades y Ciencias, (UNL). Ciudad Universitaria Paraje El pozo s/n. Santa Fe. Argentina

**Keywords:** Chelipeds, cephalothorax width, pleopods, reproduction, growth, regression model

## Abstract

The relative growth of a number of morphological dimensions of the South American freshwater crab *Trichodactylus borellianus* (Trichodactylidae) were compared and related to sexual dimorphism. Crabs were collected from ponds in the Middle Paraná River in Argentina. A regression model with segmented relationship was used to test for relative growth between these measurements where breakpoints infer the body size at which crabs reach sexual maturity. In both sexes the carapace width and the length, height, and thickness of the right and left chelae were measured, as well as the male pleopod length and the female abdomen width. All of these measurements were found to show positive allometry with the exception of the male pleopod length and the left chelae, which did not show a breakpoint. In females the breakpoint for the abdomen width inferred a morphological sexual maturity at carapace width 6.9 mm. In males the break point for the pleopod length was at carapace width 6.6 mm, with that for the chelae measurements was between carapace widths 6.4 and 6.9 mm. The relative growth pattern in *Trichodactylus borellianus* was found to be similar to that recorded for other species of the family Trichodactylidae.

## Introduction

The onset of maturity in crustaceans is signaled by a series of morphological, physiological and behavioral transformations through which immature individuals become able to produce reproductive cells and copulate (Hartnoll 1969). When organisms grow some of their morphological dimensions increase at a different rate from others that results in a change in body proportions known as relative growth (Hartnoll 1978). Relative growth data have been widely used to predict the onset of morphometric sexual maturity in a number of organisms (Haley 1969). Here we report on the results of a similar study on a species of South American freshwater crab of the family Trichodactylidae.

The Trichodactylidae is a neotropical family of freshwater crabs found throughout the river basins of tropical and subtropical South America, with the exception of the Pacific slope rivers (Magalhães 2003). Morphological maturity in the Trichodactylidae can be estimated by taking measurements of the characters of secondary sexual characters such as the male pleopods (gonopods), male chelipeds, and the female abdomen. External morphological changes that signal sexual maturity in freshwater crabs can be used to determine whether crabs are adult or immature (Somerton 1980, Somerton and MacIntosh 1983, Conan and Comeau 1986). This knowledge has been used by researchers to both identify species and to describe population structure and dynamics (Haley 1969, Hartnoll 1978, Vannini and Gherardi 1988, Felder and Lovett 1989, Chow and Sandifer 1991, Pinheiro and Fransozo 1998, Robertson and Butler IV 2003, Lima et al. 2013).

There are only a few recent studies of the allometric changes associated with growth within the genus *Trichodactylus* (Venâncio and Leme 2010, Lima et al. 2013, Silva et al. 2014). The present work focuses on allometric growth in *Trichodactylus borellianus* Nobili, 1896, which is a common species associated with the aquatic vegetation of the Paraná alluvial system in Argentina where it occurs in the Paraná and Paraguay rivers (Collins et al. 2007), as well as in Brazil (Pará, Mato Grosso, Mato Grosso do Sul), Bolivia, and Paraguay (Magalhães 2003). Previous studies on *Trichodactylus borellianus* have analyzed the influence of temperature on growth (Renzulli and Collins 2000), but the allometric changes associated with growth and the size at which it reaches sexual maturity are still unknown. A knowledge of the allometric growth of a species can be an important tool for assessing geographical variation in widespread species as *Trichodactylus borellianus* (see Hines 1989). The aim of the present study was to describe allometric growth and to estimate the onset of morphological sexual maturity of *Trichodactylus borellianus* using a series of body dimensions, including those associated with secondary sexual characters.

## Mmethods

Monthly collections were carried out during the day for fourteen months from August 2001 to October 2002. The samples were taken from three sites of the Paraná alluvial valley: Las Sandias Stream (S31°41'15.3"W 60°31'31.6"), Aliviador Stream (S31°40'17.9", W60°34'45.9") and Santa Fe River (S 31°38'35", W60°40'05.5"). The latter river is the biggest channel of La Plata Basin, containing 85% of the total freshwater in Argentina. The Paraná River system consists of a main channel with an alluvial valley ranging from approximately 13 to 56 km in width with a slope of 0.036 m km^-1^. The main channel is located on the eastern margin and is between 2 and 4.2 km wide, and is 2.3 km wide at the study site. The western area of the river has several secondary rivers, streams, ponds and islands, extending approximately 10 km in width to the main channel. The flow along the main channel varies between 10,600 m^-3^s^-1^ and 31,000 m^-3^s^-1^ causing a primary runoff in spring and summer (November to March), which originates annual floods. Autumn and winter are the low water seasons (Bonetto and Wais 1995).

**Figure 1.**
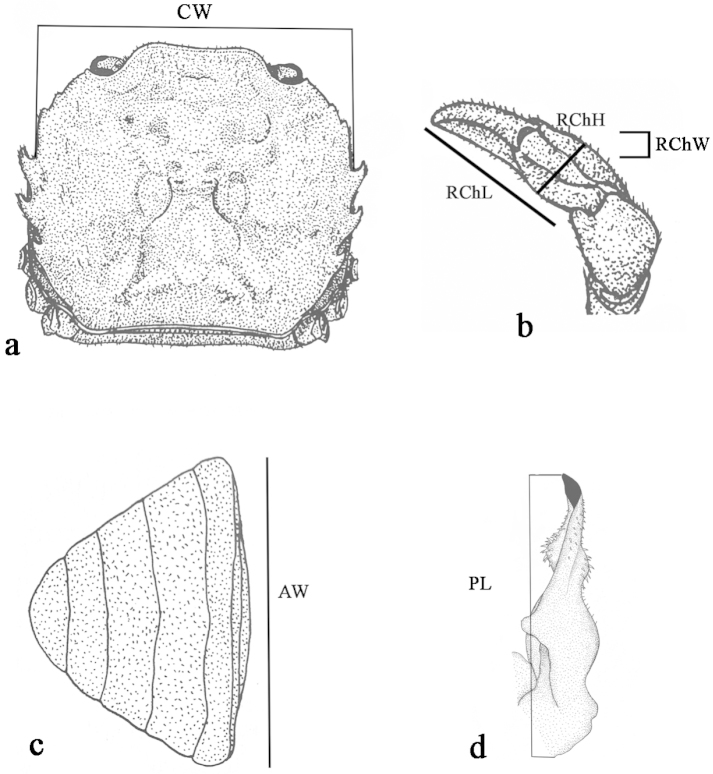
Measures taken of the crab *Trichodactylus borellianus* (modified from Magalhães 2003): **a** carapace width (CW) **b** right cheliped length (RChL), right cheliped height (RChH) and right cheliped width (RChW) **c** third abdominal width of females (AW) **d** left pleopod length (PL).

In the laboratory, a total of 337 crabs were analyzed of which 155 were males and 182 females. The following variables were recorded: carapace width (CW), right cheliped length (RChL), right cheliped height (RChH) and right cheliped width (RChW), left cheliped length (LChL), left cheliped height (LChH) and left cheliped width (LChW), abdomen width (AW) in females, and left first pleopod length (PL) in males (Fig. 1; Table 1). Organisms were measured with digital calipers to the nearest 0.01 mm, and only intermoult-stage organisms were used. Specimens with injured chela, with regenerating or missing chelipeds, or injury and/or malformation in other structures were excluded.

**Table 1.** Characterization of how the morphometric variables were obtained. CW: carapace width, ChL: cheliped length, ChH: cheliped height, ChW: cheliped width, AW: abdomen width, PL: pleopod length.

Body part (morphometric variable)	Measuring mode
CW	Distance between the first postorbital spines of the carapace
ChL	Ventral distance between distal end of the propodus and the carpus articulation
ChH	Maximum propodus thickness
ChW	Maximum distance between lateral margins of the propodus
AW	Maximum distance of the third segment of female abdomen
PL	Maximum length of the left pleopod

The CW was used as the independent variable to test relative growth in the other measurements (the dependent variables). Linear regressions, comparisons of slopes, and calculations of the onset of morphological sexual maturity were made with the software R version 2.13.2 (R development core team 2011). To analyze the onset of morphological sexual maturity a Regression Model with Segmented Relationship with breakpoint with the package ‘segmented’ was used in the regressions that visualized possible breakpoints (Muggeo 2003, 2008). This model is based on regression models where the relationships between the response and one or more explanatory variables are piecewise linear, namely represented by two or more straight lines connected at breakpoints (Muggeo 2003). Package segmented estimates linear and generalized linear models having one or more segmented relationships in the linear predictor. Estimates of the slopes and of the possibly multiple breakpoints are provided. The package includes testing/estimating functions and methods to print, summarize, and plot the results (Muggeo 2008).

The Davies’ test was used to test for significant differences of slopes between juveniles and adults and to test for a non-constant regression parameter in the linear predictor (Davies 1987). The slopes of the first segment (juveniles) and second segment (adults), when the Davies’ test was significant were estimated with the results summary of the Regression Model with Segmented Relationship with breakpoint (Muggeo 2003). A slope value = 1 represents isometric growth, a slope value < 1 represents negative allometric growth, and a slope value > 1 represents positive allometric growth.

## Results

**Figure 2.**
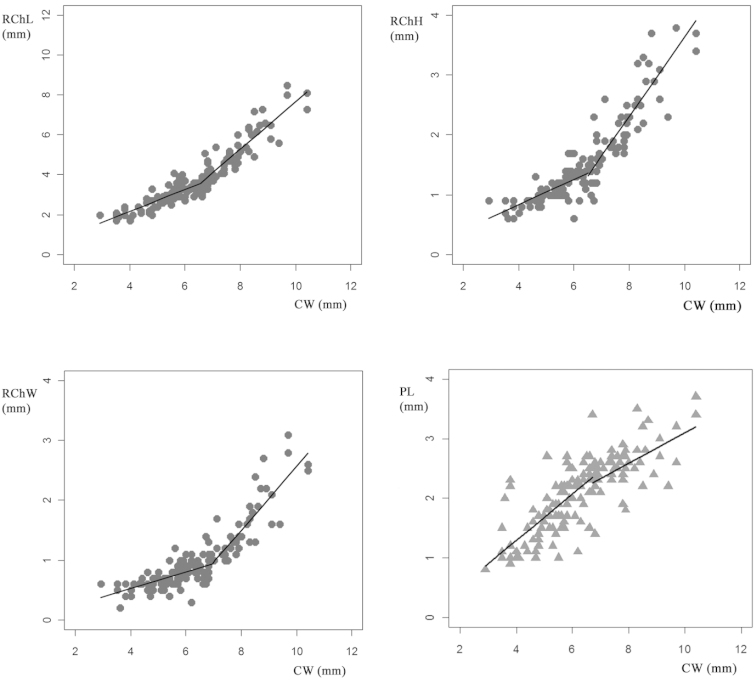
Segmented Relationship with breakpoint on male crabs of *Trichodactylus borellianus*. RChL: right cheliped length; RChH: right cheliped height; RChW: right cheliped width; PL: left pleopod length.

Male crabs had a CW of 6.34 ± 1.48 mm (ranging from 2.9 to 10.4 mm CW); female crabs had a CW of 6.12 ± 1.92 mm (ranging from 2.7 to 12.4 mm). There were no statistically significant differences between the CW of male and female crabs (t= -1.21; *p* = 0.227). The regression using CW as the independent variable indicated that measures of the right cheliped of male crabs were best adjusted to two straight lines rather than to one because these presented statistically significant differences between the slopes of both lines (Fig. 2, Table 2). However, a breakpoint was found on PL (Fig. 2) despite it not being well adjusted to the regression model and the Davies’ test finding no statistical significance (Table 2). The breakpoints produced by segmented regression ranged from CWs 6.5 to 6.9 mm for the measurements of the right chelipeds. The onset of morphological maturity size (breakpoint) for PL was similar to RChL (~CW 6.5 mm) (Table 2) (Fig. 2). No observed possible breakpoints were found for the regressions of the left cheliped in male crabs (Table 2). Juvenile and adult stages of male crabs showed negative allometric relationships between RChH, RChW, PL and CW. The slopes of the adult measurements of the right cheliped were higher than those of juveniles, and were lower in adults than juveniles for PL (Table 2, Fig. 2). The RChL vs. CW on adult male crabs showed positive allometric relationships in contrast with juveniles that presented negative allometric relationships (Table 3). Adult crabs were better adjusted to the regressions than juvenile crabs (Table 2).

**Table 2.** Results of Regression Model with Segmented Relationship with breakpoint studied for males and females of *Trichodactylus borellianus* with Davies' test for change in the slope, This includes the estimate slopes for J: juveniles and A: adults.

Sexes	Relationship	Estimated break point CW (mm)	R-squared	Intercept	Davies' test for change in the slope p-value	Stage	Slopes	Allometry
Males	RChL vs. CW	6.6	0.89	-0.02	<0.0001	J	0.55	-
						A	1.19	+
	RChH vs. CW	6.5	0.85	-0.01	<0.0001	J	0.21	-
						A	0.66	-
	RChW vs.CW	6.9	0.82	-0.02	<0.0001	J	0.14	-
						A	0.54	-
	PL vs. CW	6.6	0.60	-0.24	0.2836	J	0.39	-
						A	0.25	-
	LChL vs. CW	(*)	0.81	0.89	without slope change	-	-	
	LChH vs. CW	(*)	0.73	0.25	without slope change			
	LChW vs. CW	(*)	0.53	0.19	without slope change			
Females	RChL vs. CW	6.0	0.90	0.66	<0.0001	J	0.37	-
						A	0.62	-
	RChH vs. CW	(*)	0.85		without slope change			
	RChW vs.CW	(*)	0.70		without slope change			
	AW vs. CW	6.9	0.91	-0.79	<0.0001	J	0.73	-
						A	1.30	+
	LChL vs. CW	5.7	0.91	0.54	<0.0001	J	0.37	-
						A	0.68	-
	LChH vs. CW	(*)	0.82		without slope change			
	LChW vs. CW	(*)	0.66		without slope change			

**Figure 3.**
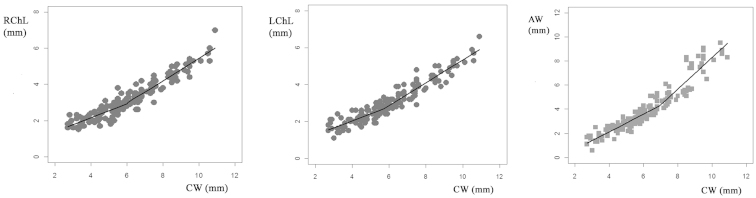
Segmented Relationship with breakpoint on female crabs of *Trichodactylus borellianus*. RChL: right cheliped length; LChL: left cheliped length; AW: third abdominal width.

In females, there were three characters that best adjusted to two straight lines rather than to one: the lengths of both chelae and the abdomen width (Table 2, Fig. 3). These presented statistically significant differences between the slopes of both lines (Table 2). Possible breakpoints were not visualized on the other measures of right and left chelipeds of female crabs (Table 2). Segmented regressions produced a breakpoint at 6.02 mm CW for RChL and a lower breakpoint for LChL (5.7 mm CW). The breakpoint of AW was somewhat higher than the cheliped lengths (Table 2). The regressions of juveniles and adult female crabs showed negative allometric relationships for RChL and LChL, and the slopes of adult measurements were higher than those of juveniles (Fig. 3, Table 3). The AW of adults showed a positive allometric relationship with CW in contrast to juveniles that presented negative allometric relationships (Fig. 3, Table 3).

**Table 3.** Results of allometric relationship study of each stage of *Trichodactylus borellianus*. J: juveniles; A: adults.

Sexes	Relationship	Stage	Linear equation *y= a + bx*	R-squared	Allometry
Males	RChL vs. CW	J	RChL = -0.0179 + 0.5478 CW	0.66	–
		A	RChL = -4.2291 + 1.1899 CW	0.80	+
	RChH vs. CW	J	RChH= -0.0109 + 0.2117 CW	0.50	–
		A	RChH = -3.003 + 0.6643 CW	0.76	–
	RChW vs. CW	J	RChW = -0.0217 + 0.1378 CW	0.40	–
		A	RChW = -2.7944 + 0.537 CW	0.68	–
	PL vs. CW	J	PL = -0.3109 + 0.3938 CW	0.46	–
		A	PL = 0.8324 + 0.2228 CW	0.28	–
Females	RChL vs. CW	J	RChL = 0.6661 + 0.3747 CW	0.59	–
		A	RChL = -0.7479 + 0.6187 CW	0.84	–
	AW vs. CW	J	AW = -0.7905 + 0.7309 CW	0.79	–
		A	AW = -4.7357 + 1.3027 CW	0.73	+
	LChL vs. CW	J	LChL = 0.5427 + 0.3747 CW	0.56	–
		A	LChL = -0.9007 + 0.6268 CW	0.87	–

## Discussion

The knowledge of the life history of a species involves understanding such aspects as the development of sexual maturity, changes in allometric growth, and the age at which each of these occur. In crabs, morphological maturity is often observed together with allometric changes in growth (González-Gurriarán and Freire 1994, Fernández-Vergaz et al. 2000).

The results obtained in this study are in line with the points proposed by Hartnoll (1978) who showed that many crustaceans, particular brachyuran crabs, have differences in relative growth. For *Trichodactylus borellianus* we also found this pattern, being able to identify male and female body parts that show morphological sexual maturity. Hartnoll (1974) believed that the growth of secondary sexual characters in crustaceans could be modeled by the power function. It was shown in this contribution that the relative growth rate differs between adults and juveniles, and that size at morphometric maturity can be estimated as the body size where the value changes. Also, in accordance with Hartnoll (1969), the population analyzed here shows that males and females maintain the same size, which favors the formation of mating pairs. The similarities in size between the sexes were also recorded for two other species of the same family (Mansur et al. 2005, Pinheiro and Taddei 2005). However, in geographically distant populations of *Trichodactylus fluviatilis* the size of males and females showed differences, while in other populations there was no size difference (Lima et al. 2013, Silva et al. 2014).

For males, the three measurements around the right cheliped showed a good fit to the regression model, as well as differences in slopes of the regressions. These three measurements revealed a similar size from which growth patterns morphometrically change. According to Bueno and Shimizu (2009), the mean values of these three estimated measures can be adopted to represent the size at the onset of morphometric maturity, being in this case 6.6 mm CW. Otherwise, Davies’ values for the PL were not significant and the R-squared values were not very high so this measure does not indicate a change in relative growth (Fig. 2d). Perhaps the pleopod width could be a better measure to indicate allometric growth than PL in this crab species. Cheliped measurements have been evaluated in many species as an indicator of relative growth and morphological sexual maturity. In males the cheliped, as an indicator of sexual maturity, could be a function of reproductive behavior (Mariappan et al. 2000, Shuster 2007). Crabs must become both functionally and physiologically mature before they can reproduce, and the ability to mate may depend on the attainment of morphometric maturity (Conan and Comeau 1986). While the reproductive behavior of this species has not yet been described, it can be suggested that *Trichodactylus borellianus* could respond to patterns of other freshwater species. Thus, according to the proposal of Gherardi and Micheli (1989), this species could be mate in intermoult, and robust chelipeds in males would be needed to support females. On the other hand, it would be interesting to consider in future investigations the possibility of the existence of agonistic behavior in this freshwater crab.

Similar relative growth patterns were registered in other trichodactylids such as *Dilocarcinus pagei*, *Sylviocarcinus australis* and *Trichodactylus fluviatilis* (Mansur et al. 2005, Pinheiro and Taddei 2005, Lima et al. 2013, Silva et al. 2014). For *Trichodactylus borellianus*, although the size range showing regression models are similar for both sexes, females show some traits that estimate maturity in different sizes. In this case, if the selected body part is LChL, females change growth patterns earlier than males. This pattern was also recorded in other brachyuran species (Negreiros-Fransozo et al. 2003, Lima et al. 2013). The size obtained with the regression model using AW vs. CW relationship is closer to the minimum size of ovigerous female (7.3 mm CW) recorded for this species (Senkman et al. in press). However, these records of ovigerous females may be underestimated due to the habits of these crabs that remain a long time in shelters and in roots of floating macrophytes (Magalhães 1986, Collins et al. 2006). Measuring the abdomen width as an indicator of relative growth and feature of morphological sexual maturity would have a relation with the incubation of eggs and the maternal care. The function of the abdomen in females as an incubator chamber is relevant in inland water species, related to the ability to incubate the eggs for a long time and to shelter the newly hatched juveniles until independence (Viau et al. 2006). For *Trichodactylus borellianus* the capacity of the abdomen as an incubator chamber increased with increasing carapace width was demonstrated by Senkman et al. (in press).

Against this background, the changes in growth shown for the equations provide evidence of the reproductive function of the abdomen width in females and the chela in males. As in the crab *Trichodactylus fluviatilis*, the relationship between RChL vs. CW also showed a positive allometry in both sexes (Lima et al. 2013, Silva et al. 2014). Allometry simply implies that the relative growth between the morphometric characters being studied is not constant. Relative to body size, chelae, abdomens, and first pleopods can all grow allometrically. Juvenile-to-adult changes in the relative growth rates of these characters can be used to identify the size of morphometric maturity (Teissier 1960). From a descriptive point of view, the AW and PL indicate that each sex may reach sexual maturity at different sizes; this pattern could be a possibility for the males to fertilise older females early.

For both sexes, the differences in the size of the chelae could involve some of different issues concerning reproduction. One of them could be related to the modification of feeding options. Variations in size of the chelae may also involve the possibilities of the range expansion of trophic items. This crab, found in the area of middle Paraná River, changes natural diet with body size (Williner and Collins 2013). This suggests a change in habitat. Larger claws could aid the crabs with changes in diet and predation that might accompany this habitat change. Adult crabs consume more plant debris and invertebrates of larger size than young crabs (Williner and Collins 2013). In turn, this diet change may correspond to a need in reproductive cell production (Nagaraju 2011). These possible associations between changes in the sizes of the chelipeds and changes in habitat and trophic potential have been recorded for the stone crabs (genus *Menippe*) from west-central Florida (Gerhart and Bert 2008).

We consider that the application of the Regression Model with Segmented Relationship with breakpoint with the package ‘segmented’ is an appropriate way to determine patterns of relative growth and sexual maturity. The present study also establishes the size range corresponding to the pubertal molt in this species. In this study, one might assume that, with the exception of traits that showed differential growth rates between adults and juveniles, for the remainder of the measured characters, that a shift in allometry between juveniles and subadults occurs in a continuous, gradual manner.

Considering the present contribution to the knowledge of reproductive traits, it is now necessary to assess sexual maturity in its other dimensions, mainly from the histological perspective, with a description of ducts and sexual cells. These results show that it is necessary to inquire about the sexual and mating system of this species in order to evaluate differences in measurements and trends found in this study (Baeza and Azorey 2012). On the other hand, as behavioral observations in the natural environment are difficult, it is recommended to raise crabs in the laboratory to evaluate mate selection and mating possibilities of different sizes.

The current study also provides a starting point for addressing additional aspects relevant to allometry of *Trichodactylus borellianus*; however variations in allometry in other parts of the range of this species and among other populations still need to be evaluated.

## References

[B1] Baeza JA Asorey C (2012) Testing the role of male-male competition in the evolution of sexual dimorphism: a comparison between two species of porcelain crabs. Biological Journal of the Linnean Society 105: 548–558. doi: 10.1111/j.1095-8312.2011.01803.x

[B2] Bonetto AA Wais IR (1995) Southern South American streams and rivers. In: Cushing CE Cummins KW Minshall GW (Eds) River and Stream Ecosystems.Ecosystems of the World, Vol. 22, Elsevier Science Publishers, Amsterdam, 257–293.

[B3] Bueno SLS Shimizu RM (2009) Allometric growth, sexual maturity and adult male chelae dimorphism in *Aegla franca* (Decapoda: Anomura: Aeglidae). Journal of Crustacean Biology 29: 317–328. doi: 10.1651/07-2973.1

[B4] Chow S Sandifer PA (1991) Differences in growth, morphometric traits, and male sexual maturity among Pacific white shrimp, *Penaeus vannamei*, from different commercial hatcheries. Aquaculture 92: 165–178. doi: 10.1016/0044-8486(91)90018-3

[B5] Collins PA Giri F Williner V (2006) Population dynamics of *Trichodactylus borellianus* (Crustacea Decapoda Brachyura) and interactions with the aquatic vegetation of the Paraná River (South America, Argentina). Annales de Limnologie – International Journal of Limnology 42(1): 19–25. doi: 10.1051/limn/2006001

[B6] Collins PA Williner V Giri F (2007) Littoral Communities: Macrocrustaceans. In: Iriondo, MH Paggi JC Parma MJ (Ed.) The middle Paraná River, limnology of a subtropical wetland. Springer-Verlag, Berlin, 277–302. doi: 10.1007/978-3-540-70624-3_11

[B7] Conan GY Comeau M (1986) Functions maturity and terminal molt of male snow crab *Chionoecetes opilio*. Canadian Journal of Fisheries and Aquatic Science 43: 1710–1719. doi: 10.1139/f86-214

[B8] Davies RB (1987) Hypothesis testing when a nuisance parameter is present only under the alternative. Biometrika 74: 33–43.

[B9] Felder DL Lovett DL (1989) Relative growth and sexual maturation in the estuarine ghost shrimp *Callianassa louisianensis* Schmitt, 1935. Journal of Crustacean Biology 9: 540–553. doi: 10.1163/193724089X00566

[B10] Fernández-Vergaz V López Abellán LJ Balguerías E (2000) Morphometric, functional and sexual maturity of the deep-sea red crab *Chaceon affinis* inhabiting Canary Island waters: chronology of maturation. Marine Ecology Progress Series 204: 169–178. doi: 10.3354/meps204169

[B12] Gerhart SD Bert TM (2008) Life-history aspects of stone crabs (genus *Menippe*): size at maturity, growth, and age. Journal of Crustacean Biology 28: 252–261. doi: 10.1163/20021975-99990372

[B11] Gherardi F Micheli F (1989) Relative growth and population structure of the freshwater crabs, *Potamon potamios palestinensis*, in the Dead Sea area. Israel Journal of Zoology 36: 133–145.

[B13] González-Gurriarán E Freire J (1994) Sexual maturity in the velvet swimming crab *Necora puber* (Brachyura, Portunidae): morphometric and reproductive analyses. ICES Journal of Marine Science 51: 133–145. doi: 10.1006/jmsc.1994.1015

[B14] Haley SR (1969) Relative growth and sexual maturity of the Texas ghost crab, *Ocypode quadrata* (Fabr.) (Brachyura, Ocypodidae). Crustaceana 17: 285–297. doi: 10.1163/156854069X00637

[B15] Hartnoll RG (1969) Mating in Brachyura. Crustaceana 16: 161–181. doi: 10.1163/156854069X00420

[B16] Hartnoll RG (1974) Variation in growth pattern between some secondary sexual characters in crabs (Decapoda, Brachyura). Crustaceana 27: 131–136. doi: 10.1163/156854074X00334

[B17] Hartnoll RG (1978) The determination of relative growth in Crustacea. Crustaceana 34: 281–293. doi: 10.1163/156854078X00844

[B18] Hines AH (1989) Geographic variation in size at maturity in brachyuran crabs. Bulletin of Marine Science 45: 356–368.

[B19] Lima DJ Cobo VJ Alves DFR Barros-Alves SP Fransozo V (2013) Onset of sexual maturity and relative growth of the freshwater crab *Trichodactylus fluviatilis* (Trichodactyloidea) in south-eastern Brazil. Invertebrate Reproduction & Development 57: 105–112. doi: 10.1080/07924259.2012.689263

[B20] Magalhães C (1986) Revisão taxonômica dos caranguejos de água doce brasileiros da família Pseudothelphusidae. Amazoniana 9: 609–636.

[B21] Magalhães C (2003) Familias Pseudothelphusidae e Trichodactylidae. In: Melo GAS (Ed.) Manual de Identificação dos CrustaceaDecapoda de Água Doce do Brasil. Edições Loyola, São Paulo, 143–287.

[B22] Mansur CB Hebling NJ Souza JA (2005) Crescimento relativo de *Dilocarcinus pagei* Stimpson, 1861 e *Sylviocarcinus australis* Magalhães & Türkay, 1996 (Crustacea, Decapoda, Trichodactylidae) no Pantanal do Rio Paraguai, Porto Murtinho, Mato Grosso do Sul. Boletim do Instituto de Pesca 31: 103–107.

[B23] Mariappan P Balasundaram C Schmitz B (2000) Decapod crustacean chelipeds: an overview. Journal of Bioscience 25: 301–313. doi: 10.1007/BF02703939 10.1007/BF02703939 11022233

[B24] Muggeo VMR (2003) Estimating regression models with unknown break-points. Statistics in Medicine 22: 3055–3071. doi: 10.1002/sim.1545 12973787 10.1002/sim.1545

[B25] Muggeo VMR (2008) segmented: an R Package to fit regression models with broken-line relationships. R News 8/1: 20–25.

[B26] Nagaraju GPC (2011) Reproductive regulators in decapod crustaceans: an overview. Journal of Experimental Biology 214: 3–16. doi: 10.1242/jeb.047183 21147963 10.1242/jeb.047183

[B27] Negreiros-Fransozo ML Colpo KD Costa TM (2003) Allometric growth in the fiddler crab *Uca thayeri* (Brachyura, Ocypodidae) from a subtropical mangrove. Journal of Crustacean Biology 23: 273–279. doi: 10.1163/20021975-99990337

[B28] Pinheiro MAA Fransozo A (1998) Sexual maturity of the specked swimming crab *Aranaeus cribrarius* (Lamarck, 1818) (Decapoda, Brachyura, Portunidae), in the Ubatuba littoral, São Paulo, Brazil. Crustaceana 71: 434–452. doi: 10.1163/156854098X00536

[B29] Pinheiro MAA Taddei FG (2005) Crescimento do caranguejo de água doce, *Dilocarcinus pagei* Stimpson (Crustacea, Brachyura, Trichodactylidae). Revista Brasileira de Zoologia 22: 522–528. doi: 10.1590/S0101-81752005000300002

[B30] R Development Core Team (2011) R: A language and environment for statistical computing. R Foundation for Statistical Computing, Vienna, Austria.

[B31] Renzulli P Collins PA (2000) Influencia de la temperatura en el crecimiento del cangrejo *Trichodactylus borellianus*. FABICIB 4: 129–136.

[B32] Robertson DN Butler IV MJ (2003) Growth and size at maturity in the spotted spiny lobster, *Panulirus guttatus*. Journal of Crustacean Biology 23: 265–272. doi: 10.1163/20021975-99990336

[B33] Senkman E Williner V Negro LC König N (in press) Fecundidad y consumo de oxigeno del cangrejo dulceacuícola *Trichodactylus borellianus* (Decapoda: Trichodactylidae) en el valle aluvial del Paraná Medio (Argentina). Hidrobiologica.

[B34] Shuster SM (2007) The evolution of Crustacean mating system. In: Duffy JE Thiel M (Eds) Evolutionary ecology of social and sexual systems. Oxford University Press, New York, 29–47. doi: 10.1093/acprof:oso/9780195179927.003.0002

[B35] Silva TR Rocha, SS Costa Neto EM (2014) Relative growth, sexual dimorphism and morphometric maturity of *Trichodactylus fluviatilis* (Decapoda: Brachyura: Trichodactylidae) from Santa Terezinha, Bahia, Brazil. Zoologia (Curitiba) 31: 20–27. doi: 10.1590/S1984-46702014000100003

[B36] Somerton DA (1980) A computer technique for estimating the size of sexual maturity in crabs. Canadian Journal of Fishery and Aquatic Sciences 37: 1488–1494. doi: 10.1139/f80-192

[B37] Somerton DA MacIntosh RA (1983) The size at sexual maturity of blue king crab, *Paralithodes platypus*, in Alaska. Fishery Bulletin 81: 621–628.

[B38] Teissier G (1960) Relative growth. In: Waterman TH (Ed.) The Physiology of Crustacea. Academic Press, New York, 537–560.

[B39] Vannini M Gherardi F (1988) Studies on the pebble crab, *Eriphia smithi* MacLeay 1838 (Xanthoidea Menippidae): patterns of relative growth and population structure. Tropical Zoology 1(2): 203–216. doi: 10.1080/03946975.1988.10539415

[B40] Venâncio FA Leme MHA (2010) The freshwater crab *Trichodactylus petropolitanus* (Goeldi, 1886) (Decapoda, Trichodactylidae) associated with roots of *Hedychium coronarium* Koenig (Zingiberacea). Pan-American Journal of Aquatic Sciences 5: 501–507.

[B41] Viau VE López Greco LS Bond-Buckup G Rodriguez EM (2006) Size at the onset of sexual maturity in the anomuran crab, *Aegla uruguayana* (Aeglidae). Acta Zoologica 87: 253–264. doi: 10.1111/j.1463-6395.2006.00239.x

[B42] Williner V Collins PA (2013) Feeding ecology of the freshwater crab *Trichodactylus borellianus* (Decapoda: Trichodactylidae) in the floodplain of the Paraná River, southern South America. Latin American Journal of Aquatic Research 41: 781–792. doi: 10.3856/vol41-issue4-fulltext-15

